# Towards a More Sustainable Nutrition: Complementary Feeding and Early Taste Experiences as a Basis for Future Food Choices

**DOI:** 10.3390/nu13082695

**Published:** 2021-08-04

**Authors:** Alessandra Mazzocchi, Valentina De Cosmi, Silvia Scaglioni, Carlo Agostoni

**Affiliations:** 1 Pediatric Intermediate Care Unit, Fondazione IRCCS Ca’ Granda Ospedale Maggiore Policlinico, 20122 Milan, Italy; alessandra.mazzocchi@unimi.it; 2 Department of Clinical Sciences and Community Health, University of Milan, 20122 Milan, Italy; valentina.decosmi@gmail.com; 3 Fondazione De Marchi, Department of Pediatrics, Fondazione IRCCS Ca’ Granda Ospedale Maggiore Policlinico, 20122 Milan, Italy; silviascaglioni50@gmail.com

**Keywords:** sustainability, nutrition, environment, Mediterranean Diet, New Nordic Diet, complementary feeding, nutritional habits

## Abstract

The concept of sustainable nutrition considers different fields: from human health to environmental, economic and socio-cultural aspects. Currently, in Europe, the diets that reflect the assumptions of the sustainable diet are the Mediterranean Diet and the New Nordic Diet. They both encourage the consumption of vegetable, organic and minimally processed foods, as well as regional, seasonal and Fair-Trade products, reducing the ecological impact of the production chain. These eating habits could be established starting from the prenatal period and from infancy during the complementary feeding stage, aiding children to accept of a more variable diet in terms of flavor, taste and texture. In particular, the positive parental role model is an effective method for improving a child’s diet and behaviors. Two healthy plates representing a sustainable diet in early infancy, at 6 and 24 months, are here proposed, in line with the “Planetary Health Diet” approved by the EAT-Lancet Commission. Our work aims to highlight how a sustainable diet is possible since infancy, since the introduction of solid foods.

## 1. Sustainable Nutrition: Definition and Principal Models

**Figure 1:**
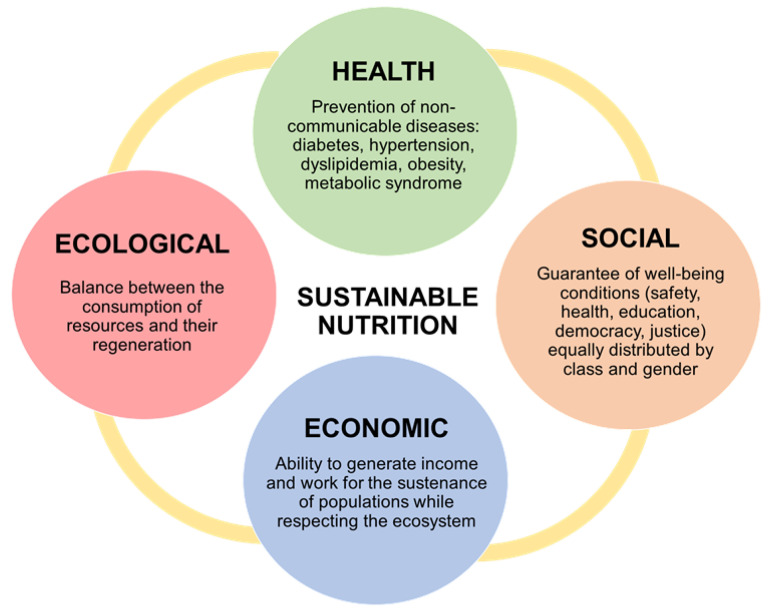
Characteristics of sustainable nutrition in the various areas.

The concept of sustainable nutrition has evolved over the years and is based on a holistic approach that now considers different fields: from human health to environmental, economic and socio-cultural aspects (Figure 1).

In 2010, the Food and Agriculture Organization of the United Nations (FAO) presented the following definition: “Sustainable Diets are those diets with low environmental impacts which contribute to food and nutrition security and healthy life for present and future generations. Sustainable diets are protective and respectful of biodiversity and ecosystems, culturally acceptable, accessible, economically fair and affordable; nutritionally adequate, safe and healthy, while optimizing natural and human resources” [1].

A safe diet should provide all nutrient and energy requirements to avoid both insufficient food supply (leading to undernutrition and communicable diseases) and overeating (leading to obesity and non-communicable diseases) [2].

Besides, the food system has a big impact on the natural environment. At the global level, the use of 48% and 70% of land and water resources derives from the food production system. Specific types of diets and eating habits contribute to greenhouse gas (GHG) emissions, affecting climate change and biodiversity in different ways [3]. The concept of “Sustainable Nutrition” considers the food supply chain at all phases, from primary production to processing, distribution, preparation, consumption and waste disposal [2].

Globalization and the growing population size led to a transition toward an urban lifestyle, accompanied by a nutritional transition. In this context, the greater food demand implies a major competition for soil, water and energy threatening food security. In addition, the trend toward the increased consumption of animal-based and processed products impacts health and the environment [4].

The leading principles of “Sustainable nutrition” are, basically: a predilection for vegetable, organic and minimally processed foods, for regional, seasonal and Fair-Trade goods, a for a reduced environmental impact of the production chain [2]

Currently, there is no unique model of a green food system. In some countries, specific dietary guidelines are available for the maintenance of public health and are affected by the local food culture, agriculture and native cuisine. In Europe, the regionally defined diets that reflect the assumptions of the sustainable diet are the Mediterranean Diet (MD) and the New Nordic Diet (NND) [5].

### 1.1. The Mediterranean Diet: Characteristic, Health and Ecological Outcomes

The MD is not a defined diet available in one single version; there are many variations adapted to the local food systems and the cultural, geographical and lifestyle contexts [3].

In general, the term “*Mediterranean diet*” denotes some common dietary characteristics including seasonal vegetables and fruits, unrefined cereals, nuts, legumes and extra virgin olive oil as the primary source of fat. This pattern implies a moderate intake of fish, dairy products and a lower to mild eating of meat, eggs, fermented beverages (mostly red wine) and sweets [6].

The literature about the favorable role of MD on physical and mental health, in particular on the risk for cardiovascular, metabolic, cancer and neurodegenerative diseases is abundant [7,8,9,10,11,12,13]

In 2010, the United Nations Educational, Scientific and Cultural Organization (UNESCO) recognized the MD as an Intangible Cultural Heritage of Humanity both for its important health and nutritional outcomes and for its environmental impact [14]. Within this definition, the MD becomes a model of sustainable diet.

This nutritional pattern has a low ecological footprint, being rich in plant-based foods [15]. Local, traditional, fresh and minimally processed foods are preferred and recommended to be consumed daily. In this way, health is preserved because products maintain a greater quantity of vitamins, minerals and other nutrients contributing to the general well-being. Furthermore, the high fiber supply confers a good satiety value and promotes body weight maintenance, blood glucose and cholesterol control [16]. At the same time, a more vegetable food production reduces the use of natural resources in terms of land and water and decreases GHG emissions, unlike livestock production. The ecological benefits derive not only from the quality of food but also from the whole food chain system, including production, transformation, distribution, cooking and disposal consumption. The preference toward plant-based, territorial, seasonable (and organic) foods aids to maintain biodiversity, preserves the landscapes and the sea and sustains the local economy [16].

Recently, a new graphical illustration of the MD pyramid has been conceived, empathizing the concept of sustainability and encouraging the consumption of biodiverse, eco-friendly and traditional foods. The aim was to highlight the role of MD for its benefits not only for a single individual, but also for the whole planet and the future generations [16].

### 1.2. The New Nordic Diet: Characteristic, Health and Ecological Outcome

The NND was developed within the Danish research project OPUS according to the same crucial values of health, gastronomic potential, Nordic identity and sustainability [17]. It is focused on the consumption of fruit and vegetables from the Nordic regions, with a particular emphasis on the health benefits of native berries, cabbages, root vegetables and legumes. It also implies a high content of fresh herbs, potatoes (not fries), wild plants and mushrooms, whole grains and nuts (unsalted and non-oiled). Lastly, regular fish consumption is recommended, alternating between fatty and lean species and between sources (Atlantic, Baltic, freshwater), but also seaweed, free-range animals and wild game [17].

Studies on the NND are mostly on adult populations and have demonstrated positive results on weight, metabolic and cardiovascular disease [18,19,20,21,22], leading to a lower total mortality [23,24]. This pattern has also been assessed in a greater crossover intervention study among Danish school children, where the results showed a better dietary intake at the food and nutrient levels when their usual meals followed the principles of the NND and beneficial effects on blood pressure, blood lipid profile and insulin sensitivity [25,26]. Lastly, in the context of identifying optimal and evidence-informed diets, a report from the World Health Organization (WHO) identified both the MD and the NND as region-specific healthy diets [27].

Within these two dietary scenarios, a recent study by Ulaszewska et al. [5] presented the “environmental hourglass (EH) approach”, trying to explain dietary advice in terms of food categories, their frequency and the portion of consumption indicated in light of their environmental consequences. The results highlighted that the total GHG emission impact was similar (23.56 Kg CO2 eq/week for the MD and 25.8 Kg CO2 eq/week for the NND).

Thus, food choices according to healthy dietary patterns as those typical of the Mediterranean basin and the Nordic countries, could be preferred also from an eco-friendlier perspective. These eating habits could be established starting from the prenatal period and from infancy, aiding children to accept a more variable diet in terms of flavor, taste and texture.

## 2. Complementary Feeding and Early-Life Experiences

### 2.1. Timing for Complementary Feeding

During the first thousand days of life, healthy eating habits should be learned, and this should be the objective of preventive policies. The WHO recommends exclusive breastfeeding for the first six months of life, as human milk provides all the energy and nutrients infants need for adequate growth, protects against infection and promotes an optimal cognitive development. From around 6 months of age, other foods besides milk must be introduced in the infant’s diet for nutritional and developmental needs [28]. The CF period is characterized by rapid growth and infants are vulnerable to nutrient deficiencies and excesses.

### 2.2. Child’s Taste Experiences

During CF, modifications in the infants’ diet occur, with the exposure to new foods, tastes and feeding experiences. The recommendations from the European Society for Pediatric Gastroenterology, Hepatology and Nutrition (ESPGHAN) state that a varied diet since the initial stages of CF should be offered to infants. Exposing infants to foods with different flavors and textures, including bitter-tasting green vegetables, is desired. Infants have innate preferences for sugar and salty savors, and refuse bitter tastes. Parents may act on these preferences, by offering foods without added sugars and salt, and by the timely presentation of a diversity of flavors [29]. Since the in-utero life, the fetus is exposed to a variety of tastes. The amniotic fluid also acts as a transporter of tastes from the mother’s diet, which stimulates the fetus’ chemosensory system. Later, when the baby is born, it will be exposed to a variety of tastes from breast milk, that reflect the composition of the maternal diet. In this way, a repeated exposure to these flavors increases infants’ acceptance of foods since the very early stages of life [30].

### 2.3. The Role of Family Environment

The period of CF is decisive, not only for the child tastes’ preferences, but also for reorganizing the dietary choices of the whole family. New consumption habits and choices may be settled during this period, shifting the choices toward more sustainable foods and toward avoiding food waste. The food choices of the family will be reflected in the choices of the young adult. For this reason, the family environment has the role to set both taste preferences and the infant’s attitude toward food, with attention to the environmental impact (and optimizing the long-term public health benefits) [31]. Populations have different habits and cultural perspectives regarding foods. In Italy, Spain, Greece and all the countries on the Mediterranean gulf, the CF starts by following the Mediterranean Diet. Otherwise, Nordic Countries start with their typical and more frequently consumed foods. Lind and colleagues began a new randomized, controlled study on Swedish infants (6 to 18 months of age), with the purpose of following them at different time points, and to compare the currently suggested Swedish complementary diet, to one based on Nordic foods. The Nordic Diet promotes a major intake of fruits, berries, vegetables, tubers, whole-grain and game, and a lower intake of sweets, dairy, meat and poultry, with a lower protein content (30% decrease), a higher intake of vegetable fats and fish and a systematic introduction of fruits and greens. Authors will collect information regarding children’s developmental maturation, biochemical values, nutritional status, eating behavior and food acceptance later in life [32]. The results the authors expect are a better body composition and a shift of metabolism toward a less obesogenic, diabetogenic and inflammatory pattern. Furthermore, the authors expect an improved gut microbiota composition, a more ecological nutrient intake and an augmented acceptance of healthy foods.

## 3. Tracking of Nutritional Habits and Cultural Values Connected to Sustainability

Changes in dietary patterns in adults have been investigated in numerous studies [33,34,35,36]. However, differences in follow-up times, sample sizes and methodologies make it difficult to draw any firm conclusions about the stability of dietary patterns over time.

Different reasons may induce changes in dietary patterns by individuals, e.g., major life happenings, variations in food preferences or in nutritional recommendation, changes in the food supply or influences of mass media and peers.

The Avon Longitudinal Study of Pregnancy and Childhood (ALSPAC) aimed to track children’s nutritional habits. Within this study, the stability of dietary patterns was evaluated on 6177 children [37]. The results identified virtually identical dietary patterns at the ages of 4 and 7 and, the emergence of periods of change between the ages of 3 and 4 and the ages of 7 and 9.

Other studies demonstrated that food preferences begin early in life and remain constant to adulthood. Food behaviors and attitudes are difficult to modify in adults and in older children [38,39].

For all these aspects, understanding how and by what food habits are influenced, is a matter of concern to contrast the formation of unhealthy dietary behaviors and the development of metabolic disorders in adulthood.

### 3.1. The Influence of Breastfeeding

Two factors are related to higher vegetable consumption during childhood: (1) longer breastfeeding [40], (2) a higher fruit and vegetable intake since infancy [41]. A sensitive period in human flavor learning exists, highlighting the importance of the age of introduction to fruit and vegetables [42].

Children’s early exposure to foods influences the development of taste and food preferences, which in turn has an impact on subsequent eating habits. Further, some aspects of diet and growth in infancy, such as breast-feeding, protein intake and rapid early weight gain, have been shown to exert an influence on adiposity [43,44,45] and poor cardiovascular health [46].

### 3.2. The Influence of Parental Role

A positive parental role model may be a better and more effective method for improving a child’s diet than attempts at dietary control [47].

The Melbourne InFANT Program underlines the importance of promoting healthy dietary trajectories focusing on infancy and involving parents. At this stage of life, the home environment modulates dietary intakes, the foods provided by parents and parental feeding practices and modeling.

Furthermore, since behaviors related to dietary intake are modifiable, the tracking of intakes for healthier food choices may have implications on public health interest [48]. Given that, the eating behavior of each individual produces effects on the sustainability of the environment, understanding the factors that facilitate or hinder sustainable eating behaviors can contribute to the improvement of effective intervention and communication plans aimed at raising consumer awareness to the importance of food choices [48].

### 3.3. The Influence of Cultural Values

A study analyzed cultural values related to sustainability from different demographic groups across Europe which make some people more inclined to sustainable behavior. The values identified to promote sustainable food choices were: altruism, willingness to contribute to the improvement of the conditions of the community in which one lives, willingness to sacrifice their income to prevent environmental pollution, a spirit of equality, trust in others, simpler and more natural lifestyle, tolerance and respect [49].

As mentioned above, the role of parents is fundamental for the well-being of the next generations. Indeed, parents should be an example and teach their children to have respect for, and take care of, their health and the planet. There is therefore a need to focus on the education of children to achieve a high social cohesion, tolerance and respect, important qualities that also indicate a great sensitivity regarding environmental and social sustainability. So why not start with complementary feeding?

### 3.4. Early Taste Experiences and Later Food Preferences

In the early stages of life, the first sensory experiences and habits interact with the genetic predisposition and are essential for the establishment of taste [50].

The learning process that began during pregnancy continues during breastfeeding and continues over the years. Food preferences are closely linked to habit. The most important determinant of a preference for a particular food is how familiar it is. Children love what they know and eat what they love. At all stages, the example of parents continues to be very important. When addressing an infant and a young child, education does not need words: it is conveyed through the repeated offer of a varied and healthy diet and example [50].

### 3.5. The Adult Approach to a Sustainable Nutrition

If parents and family heavily influence children and are essential for the development of eating habits and taste, what is the position of adults regarding sustainable nutrition and therefore the cultural environment in which children grow up?

Consumer attitudes toward eco-sustainable food behavior in Italy were studied within the REGALIM project, conducted in 2015 by the Ministry of Agricultural, Food and Forestry Policies [51].

Most of the respondents had a favorable attitude toward foods produced with eco-sustainable techniques and even more toward foods produced locally, probably because they are better known and more familiar. Freshness, authenticity and price were revealed as the most important factors determining the purchase decision, while the environmental impact was considered among the top three most important factors by only 7.2% of respondents. In conclusion, the strongly positive attitudes toward the consumption of sustainable products do not translate into the purchase or intention to buy such products [51].

## 4. Conclusions

The complementary feeding period is recognized to be a critical window for the promotion of optimal growth and of a healthy behavioral development. Many factors influence the future nutritional habits of the infant and, according to this perspective, it is important to imprint a dietary approach based also on sustainable values.

However, much still needs to be done to promote to the awareness of the importance of food choices also from the sustainability point of view.

**Figure 2:**
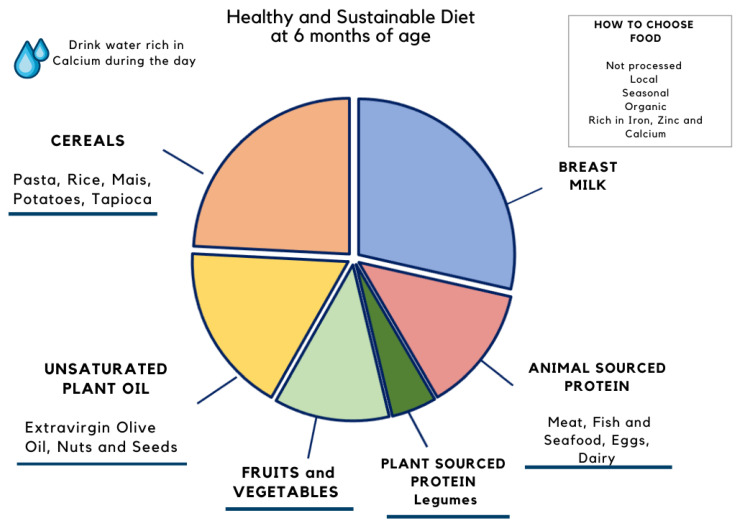
Proposal of a sustainable diet that respects EFSA nutritional recommendations for an infant of 6 months of age fed breast milk on demand and 2 complementary feeding meals. In the first year, breast milk supply should preferably provide an energy intake of between 33% and 50% of total energy.

**Figure 3:**
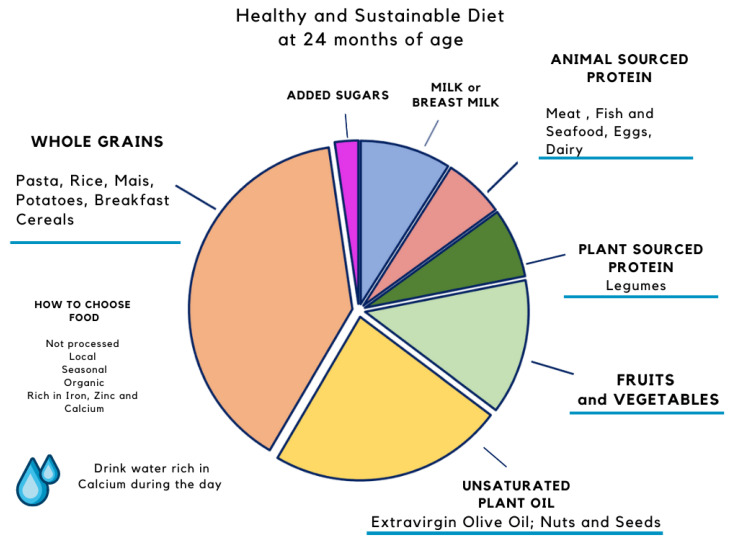
Proposal of a sustainable diet that respects EFSA nutritional recommendations for an infant at 24 months of age fed breast milk on demand and 2 complementary feeding meals. At this age, breast milk or substitutes supply should preferably provide an energy intake of between 10% and 20% of total energy.

Recently, in this context, the EAT-Lancet Commission proposed a global planetary health diet that is healthy for both people and the planet, called the “Planetary Health Diet” (PHD) [52]. Approximately half of the PHD plate would be covered with vegetables and fruit of different colors; a third made up of whole grains, followed by plant proteins (beans, pulses), some unsaturated oils with optional but modest amounts of animal protein and dairy and some added sugars and starchy. Since infants have different nutritional requirements, and it is not possible to adapt the scheme currently used for adults to their diets, we proposed schemes of healthy diets for infants aged 6 and 24 months, in Figure 2 and Figure 3, respectively.

Besides the macro and micronutrients composition of these plates, the practical advice highlighted to improve a sustainable behavior are: (1) prefer foods produced close to home, especially fruits, vegetables and legumes; (2) choose non-processed foods and with a very small amount of added ingredients; (3) prefer non-packaged meals; (4) plan accurately the shopping of the foods necessary for weekly meals.
